# Use of Proline to Induce Salt Stress Tolerance in Guava

**DOI:** 10.3390/plants13141887

**Published:** 2024-07-09

**Authors:** Smyth Trotsk de Araújo Silva, Geovani Soares de Lima, Vera Lúcia Antunes de Lima, Jackson Silva Nóbrega, Saulo Soares da Silva, Jean Telvio Andrade Ferreira, Maila Vieira Dantas, Iara Almeida Roque, Lauriane Almeida dos Anjos Soares, Rafaela Aparecida Frazão Torres, Cassiano Nogueira de Lacerda, Hans Raj Gheyi, Luderlândio de Andrade Silva, Valéria Fernandes de Oliveira Sousa

**Affiliations:** 1 Academic Unit of Agricultural Engineering, Federal University of Campina Grande, Campina Grande 58430-380, PB, Brazil; smythtrotsk18@gmail.com (S.T.d.A.S.); geovani.soares@professor.ufcg.edu.br (G.S.d.L.); vera.lucia@professor.ufcg.edu.br (V.L.A.d.L.); jeantelvioagronomo@gmail.com (J.T.A.F.); maila.vieira02@gmail.com (M.V.D.); yara.roque.sb@gmail.com (I.A.R.); rafaela.aparecida@estudante.ufcg.edu.br (R.A.F.T.); cassianonogueiraagro@gmail.com (C.N.d.L.); hans.gheyi@ufcg.edu.br (H.R.G.); 2 Postgraduate Program in Agroindustrial Systems, Federal University of Campina Grande, Pombal 58840-000, PB, Brazil; saulosoares90@gmail.com (S.S.d.S.); luderlandioandrade@gmail.com (L.d.A.S.); 3 Academic Unit of Agrarian Sciences, Federal University of Campina Grande, Pombal 58840-000, PB, Brazil; lauriane.almeida@professor.ufcg.edu.br (L.A.d.A.S.); valeriafernandesbds@gmail.com (V.F.d.O.S.)

**Keywords:** *Psidium guajava* L., water scarcity, amino acid

## Abstract

Guava is a fruit tree with high potential in the semi-arid region of northeast Brazil. However, qualitative and quantitative water scarcity is a limiting factor for the expansion of irrigated agriculture. Thus, it is necessary to use techniques to mitigate the effects of salt stress, such as foliar application of proline. The objective of this study was to evaluate the effect of foliar application of proline as a mitigator of salt stress effects on the morphophysiology of guava cv. Paluma. The experiment was carried out under field conditions at the ‘Rolando Enrique Rivas Castellón’ Experimental Farm in São Domingos, PB, Brazil, using a randomized block design in a 5 × 4 factorial scheme referring to five levels of electrical conductivity of irrigation water, ECw (0.8, 1.5, 2.2, 2.9, and 3.5 dS m^−1^) and four concentrations of proline (0, 8, 16, and 24 mM). Salinity above 0.8 dS m^−1^ compromised gas exchange, photosynthetic pigment synthesis, photochemical efficiency, and growth of guava plants at 360 days after transplanting. Foliar application of proline at a concentration of 24 mM mitigated the effect of salt stress on the relative water content, stomatal conductance, and carotenoid contents in plants irrigated with 3.6 dS m^−1^ water. Meanwhile, a proline concentration of up to 18 mM resulted in higher transpiration, CO_2_ assimilation rate, instantaneous carboxylation efficiency, and absolute growth rate in stem diameter under ECw of 0.8 dS m^−1^. Proline concentration of up to 24 mM increased the biosynthesis of photosynthetic pigments and the relative growth rate in stem diameter of guava in the period from 190 to 360 days after transplanting.

## 1. Introduction

Guava (*Psidium guajava* L.) is a species belonging to the family Myrtaceae, whose origin is located between the tropical and subtropical regions of Mexico, Central, and South America [1]. Guava fruit has great economic importance in Brazil, being consumed fresh and/or in the agro-industrial segment as pulp, ice cream, puree, and jam. In addition, it has nutritional value as a source of iron, calcium, phosphorus, potassium, ascorbic acid, and vitamin A and B vitamins, besides being rich in fiber [2].

Brazil ranks third in guava production in the world, with a total of 567,764 tons being produced in the 2022 season in a harvested area of 22,684 hectares, obtaining an average production per hectare of almost 25 thousand tons. Pernambuco and São Paulo stand out among the largest guava-producing states [3]. The expansion of guava cultivation areas in the northeast occurred mainly through the quick financial return of the capital invested and due to it being a versatile fruit in its forms of use; as such, guava represents a real alternative in the development of the fruit-growing sector of the northeast [4].

In 2022, the state of Paraíba produced only 2557 tons in an area of 338 hectares, and its yield was lower than those of the other guava-producing states [3]. The limitation of guava production is related to irregular rainfall associated with high temperatures and evapotranspiration rates, making the use of irrigation indispensable for safe production [5]. The water sources available for irrigation in the Brazilian semi-arid region, with some exceptions, contain high levels of adsorbed salts, predominantly chloride and sodium [6].

High concentrations of salts in water and/or soil represent one of the abiotic stresses that most limit crop production [7] due to the osmotic and ionic effects, which hinder the absorption of water and nutrients and cause specific toxicity due to the high concentration of phytotoxic ions (Na^+^ and Cl^−^) in the cytoplasm, nutritional imbalance, and physiological and metabolic changes, such as oxidative stress, resulting in changes in the cell structure and the rupture of membranes [8,9].

However, the intensity of salt stress effects on plants varies depending on the cationic nature of the water, the time of exposure to stress, the genotype, the developmental stage, irrigation management, fertilization, climatic conditions, and the application of exogenous substances [10,11]. The guava cultivar Paluma in its initial stage of development is sensitive to irrigation water salinity, with a threshold ECw of 0.3 dS m^−1^ [12] and 2.15 dS m^−1^ in the post-grafting stage [13].

Considering the socioeconomic importance of guava cultivation in the northeast region of Brazil, it is necessary to use strategies capable of mitigating the harmful effects of salt stress on this fruit crop. Several strategies have been used to mitigate the effects of salt stress on plants, including foliar application of proline [14,15,16]. Under conditions of abiotic stress, proline can act as a signaling and/or regulatory osmolyte that activates multiple physiological, biochemical, and molecular responses [17], and it can be used in the stabilization of membranes and proteins, the scavenging of reactive oxygen species (ROS), the maintenance of intracellular pH, acting on molecular signaling, oxidation reduction balancing, and the induction of gene expression [18].

Several studies have been conducted to evaluate the effects of foliar application of proline in mitigating salt stress effects on plants, such as guava in the seedling formation stage. Veloso [19] concluded that foliar application of proline reduced the harmful effects of water salinity on seedling growth. Gohari [20], in grapevines, found that foliar application of proline improved growth, photosynthetic pigment synthesis and antioxidant activity under saline conditions. However, studies evaluating the effects of foliar application of proline in guava under irrigation with saline waters throughout the development cycle are scarce in the literature. Thus, it is extremely important to conduct research with the purpose of evaluating mitigation strategies with foliar application of proline in guava under irrigation with waters of different salinity levels.

In this context, the hypothesis of this study is that foliar application of proline increases the tolerance of guava plants through the elimination of ROS and by aiding in the accumulation of osmoprotective substances, thus attenuating the deleterious effects of salt stress on gas exchange, water status, pigment synthesis, and photochemical efficiency supporting the growth of guava cv. Paluma.

The objective of this study was to evaluate the effect of foliar application of proline as a mitigating factor of salt stress on the morphophysiology of guava cv. Paluma.

## 2. Results

### 2.1. Relative Water Content, Electrolyte Leakage in the Leaf Blade, and Water Saturation Deficit

According to the summary of the analysis of variance (Table 1), there was a significant effect (*p* ≤ 0.01) of the interaction between the electrical conductivity of irrigation water (ECw) and proline concentrations (PROL) on the relative water content (RWC), water saturation deficit (WSD), and electrolyte leakage (EL%) in the leaf blade of guava plants cv. Paluma at 360 days after transplantation (DAT).

**Figure 1:**
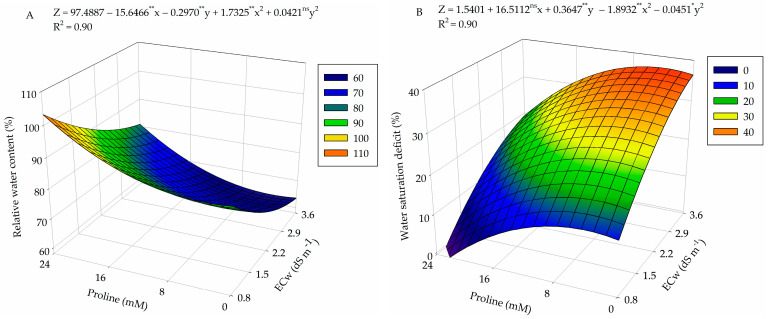

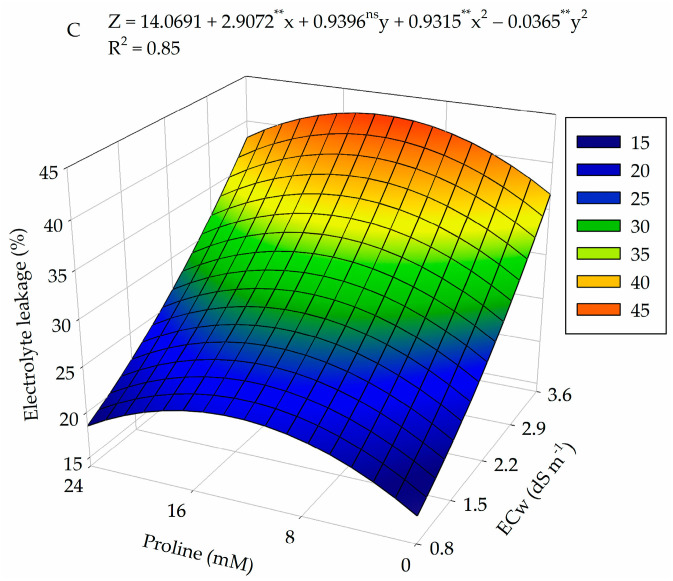
Relative water content (RWC) (**A**), water saturation deficit (WSD) (**B**), and electrolyte leakage (EL%) (**C**) in the leaf blade of guava plants as a function of the interaction between electrical conductivity levels (ECw) and proline concentrations at 360 days after transplanting. X and Y: levels of electrical conductivity of water (ECw) and proline concentrations (PROL), respectively. ** and * significant at *p* ≤ 0.01 and *p* ≤ 0.05 and ^ns^ not significant (*p* > 0.05) according to the F test, respectively.

Foliar application of proline mitigated the effect of salt stress on the relative water content (RWC) in the leaf blade, with the highest estimated value (100%) obtained in plants subjected to ECw of 0.8 dS m^−1^ and a proline concentration of 24 mM (Figure 1A). In contrast, the lowest value (63.6%) occurred in plants cultivated under an estimated water salinity of 3.0 dS m^−1^ and that did not receive foliar application of proline. 

Water saturation deficit (WSD) in the leaf blade was higher (37.17%) in plants subjected to ECw of 3.6 dS m^−1^ and foliar application of proline at a concentration of 4.5 mM (Figure 1B). However, foliar application of proline reduced WSD in plants irrigated using water with electrical conductivity of 0.8 dS m^−1^, and a value of 0.79% was observed in plants that received proline application at a concentration of 24 mM. 

Electrolyte leakage (EL%) in the leaf blade of guava increased with the increase in irrigation water salinity, with the highest value (42.6%) reached in plants subjected to ECw of 3.6 dS m^−1^ and under a proline concentration of 13.5 mM (Figure 1C). Conversely, the lowest EL% value (17%) occurred in plants cultivated under the lowest ECw level (0.8 dS m^−1^) and in plants that did not receive foliar application of proline (0 mM), indicating that the increase in salt stress promoted a 25.6% increase when comparing the maximum value obtained at ECw of 3.6 dS m^−1^ to that obtained at the lowest salinity level (0.8 dS m^−1^).

### 2.2. Gas Exchange

There was a significant effect of the interaction between the factors electrical conductivity of irrigation water (ECw) and proline concentrations (PROL) on the stomatal conductance, transpiration, internal CO_2_ concentration, CO_2_ assimilation rate, instantaneous carboxylation efficiency, and instantaneous water use efficiency of guava plants (Table 2).

**Figure 2:**
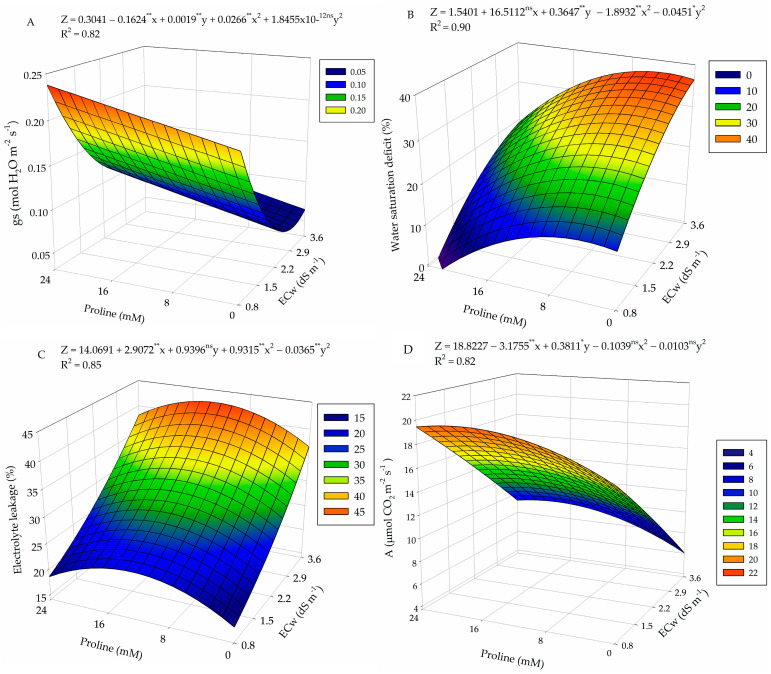

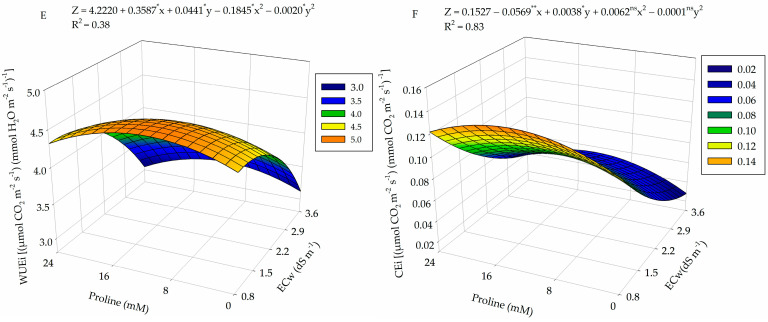
Stomatal conductance (gs) (**A**), transpiration (E) (**B**), internal CO_2_ concentration (Ci) (**C**), CO_2_ assimilation rate (A) (**D**), instantaneous water use efficiency (WUEi) (**E**), and instantaneous carboxylation efficiency (Cei) (**F**) of guava plants cv. Paluma as a function of the interaction between levels of electrical conductivity of water (ECw) and proline concentrations at 360 days after transplanting. X and Y: levels of electrical conductivity of water (ECw) and proline concentrations, respectively. ** and * significant at *p* ≤ 0.01 and *p* ≤ 0.05 and ^ns^ not significant (*p* > 0.05) according to the F test, respectively.

For stomatal conductance (Figure 2A), plants grown under a proline concentration of 24 mM and irrigation water electrical conductivity of 0.8 and 3.6 dS m^−1^ obtained higher values (0.24 and 0.11 mol H_2_O m^−2^ s^−1^), respectively. The lowest value of 0.05 mol H_2_O m^−2^ s^−1^ was reached in plants subjected to ECw of 2.9 dS m^−1^ without proline application (0 mM).

For the transpiration (E) of guava plants (Figure 2B), the highest value obtained (19.73 mmol H_2_O m^−2^ s^−1^) occurred in plants subjected to ECw of 0.8 dS m^−1^ and under foliar application of proline at a concentration of 18 mM. On the other hand, the lowest value (6.04 mmol H_2_O m^−2^ s^−1^) of E was observed in plants subjected to the highest salinity (3.6 dS m^−1^) and that did not receive proline application, which resulted in a decrease of 69.3% when comparing the maximum and minimum values obtained.

Regarding internal CO_2_ concentration, the maximum value of 252.27 μmol CO_2_ m^−2^ s^−1^ was observed in guava plants irrigated using water with electrical conductivity of 3.6 dS m^−1^ and foliar application of proline at a concentration of 24 mM (Figure 2D). In contrast, the minimum value of 131.43 μmol CO_2_ m^−2^ s^−1^ was observed in plants under ECw of 0.8 dS m^−1^ and an estimated proline concentration of 9.0 mM. When comparing the maximum and minimum values obtained, a decrease of 47.9% was observed.

Regarding the CO_2_ assimilation rate (Figure 2D), plants irrigated using water with electrical conductivity of 0.8 dS m^−1^ and foliar application of proline at a concentration of 18 mM obtained the highest value (19.73 μmol CO_2_ m^−2^ s^−1^). The lowest value obtained was 6.04 μmol CO_2_ m^−2^ s^−1^, which was reached in plants irrigated with ECw of 3.6 dS m^−1^ and without proline application (0 mM). 

Instantaneous water use efficiency (WUEi) was higher (4.64 [(μmol CO_2_ m^−2^ s^−1^) (mmol H_2_O m^−2^ s^−1^)^−1^]) when plants were subjected to ECw of 0.98 dS m^−1^ and a proline concentration of 10.5 mM (Figure 2E). However, the lowest WUEi (3.05 [(μmol CO_2_ m^−2^ s^−1^) (mmol H_2_O m^−2^ s^−1^)^−1^]) was observed in plants irrigated with 3.6 dS m^−1^ water and under foliar application of proline at a concentration of 24 mM, which represents a reduction of 34.3% when compared to the highest value obtained.

For instantaneous carboxylation efficiency (Figure 2F), the electrical conductivity of water of 0.8 dS m^−1^ and an estimated proline concentration of 13.5 mM resulted in a maximum value of 0.13 [(μmol CO_2_ m^−2^ s^−1^) (μmol CO_2_ m^−2^ s^−1^)^−1^]. The minimum value of 0.02 [(μmol CO_2_ m^−2^ s^−1^) (μmol CO_2_ m^−2^ s^−1^)^−1^] was found in plants without proline application (0 mM) and under ECw of 3.6 dS m^−1^.

### 2.3. Photosynthetic Pigments

There was a difference between the salinity levels, with a significant effect (*p* ≤ 0.01) on the contents of chlorophyll *a*, chlorophyll *b*, total chlorophyll, and carotenoids of guava cv. Paluma (Table 3). Proline concentrations significantly influenced (*p* ≤ 0.01) all variables related to photosynthetic pigments. Regarding the interaction between the factors (ECw × PROL), a significant effect was observed only in carotenoids (*p* ≤ 0.01) of guava cv. Paluma at 360 days after transplanting

**Figure 3:**
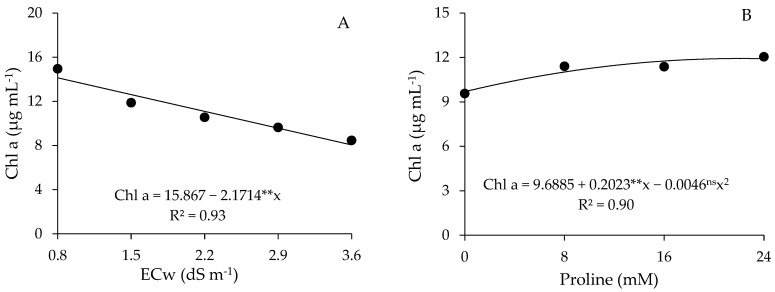

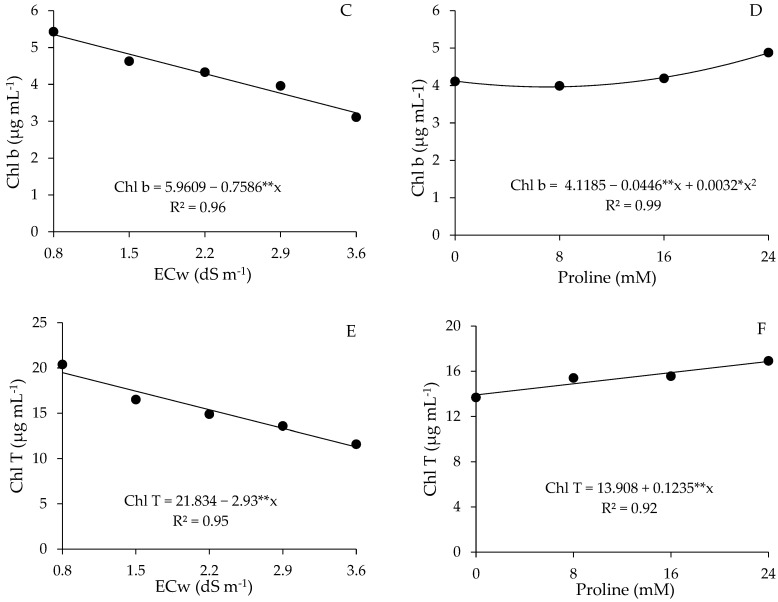
Contents of chlorophyll *a* (Chl a), chlorophyll *b* (Chl b), and total chlorophyll (Chl T) of guava plants cv. Paluma as a function of the levels of electrical conductivity of the water (ECw) (**A**,**C**,**E**) and proline concentrations (**B**,**D**,**F**) at 360 days after transplanting. ** and * significant at *p* ≤ 0.01 and *p* ≤ 0.05 and ^ns^ not significant (*p* > 0.05) according to the F test, respectively.

Irrigation with saline water significantly affected the synthesis of photosynthetic pigments, and it was observed for the contents of chlorophyll *a*, chlorophyll *b*, and total chlorophyll (Figure 3A–C, respectively) that the plants had the highest values at ECw of 0.8 dS m^−1^ with the increase in salinity levels, with reductions of 43.0, 39.7, and 42.1%, respectively, when comparing the values obtained in plants irrigated with water of 3.6 dS m^−1^ to those of plants subjected to ECw of 0.8 dS m^−1^. 

Foliar application of proline positively influenced the contents of chlorophyll *a*, chlorophyll *b*, and total chlorophyll of guava cv. Paluma. For Chl *a* and Chl *b* contents, a quadratic response was observed (Figure 3B,D), with maximum values of 12, 04, and 4.88 μg mL^−1^, respectively. For the total chlorophyll contents (Figure 3F), it was observed that foliar application of proline resulted in a linear increase equal to 0.88% per unit increase in proline concentration. When comparing plants that received the highest proline concentration (24 mM) to those in the control treatment (0 mM), an increase of 2.46% was observed in Chl *T* contents.

**Figure 4:**
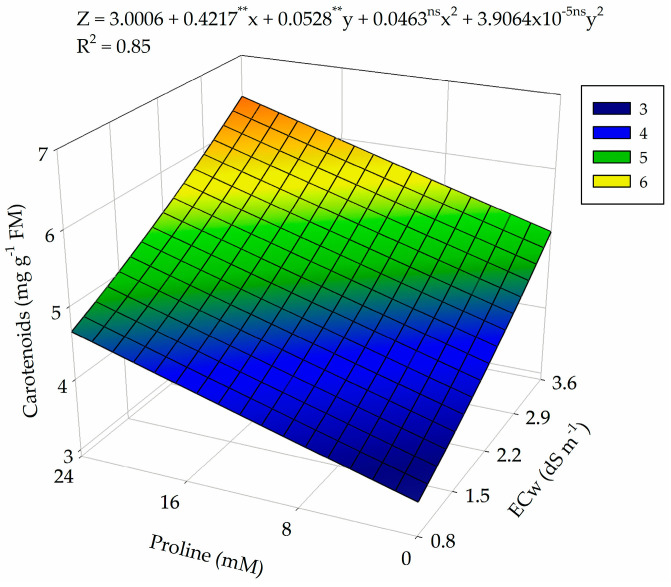
Carotenoid content of guava plants as a function of the interaction between levels of electrical conductivity of water (ECw) and proline concentrations at 360 days after transplanting. X and Y: levels of electrical conductivity of water (ECw) and proline concentrations, respectively; ** and * significant at *p* ≤ 0.01 and *p* ≤ 0.05 and ^ns^ not significant (*p* > 0.05) according to the F test, respectively.

The increase in the electrical conductivity of irrigation water and proline concentrations stimulated the synthesis of carotenoids in guava cv. Paluma (Figure 4), with the highest content (6.25 m μg mL) observed in plants subjected to the highest ECw level (3.6 dS m^−1^) and a proline concentration of 24 mM. On the other hand, the lowest value (3.36 μg mL^−1^) was found in plants subjected to ECw of 0.8 dS m^−1^ and that did not receive foliar application of proline (0 mM). When comparing the Car contents, it was observed that the increase up to the highest ECw and a proline concentration of 24 mM promoted an increase of 46.2% in comparison to the values obtained at ECw 0.8 dS m^−1^ and a proline concentration of 0 mM. 

### 2.4. Photochemical Efficiency

There was a significant effect (*p* ≤ 0.05) of salinity levels (ECw) and proline concentrations (PROL) on the initial fluorescence (F_0_) of guava cv. Paluma (Table 4). In contrast, the water salinity levels significantly affected the maximum fluorescence (F_m_) and variable fluorescence (F_v_) of guava plants cv. Paluma at 360 days after transplanting.

**Figure 5:**
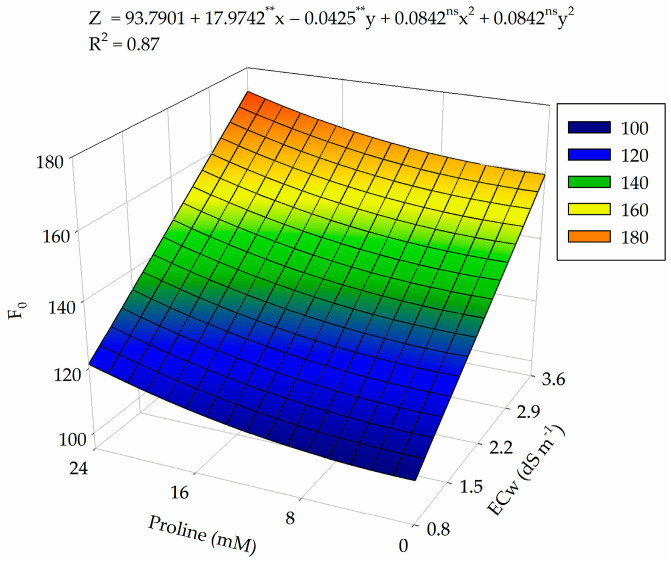
Initial fluorescence (F_0_) of guava plants cv. Paluma as a function of the interaction between levels of electrical conductivity of water (ECw) and proline concentrations at 360 days after transplanting. X and Y: levels of electrical conductivity of water (ECw) and proline concentrations, respectively; ** and * significant at *p* ≤ 0.01 and *p* ≤ 0.05 and ^ns^ not significant (*p* > 0.05) according to the F test, respectively.

For the initial fluorescence (F_0_), the increase in water salinity and proline concentrations promoted an increase in F_0_ (Figure 5), with the highest value (172.66) obtained in plants irrigated with ECw of 3.6 dS m^−1^ and under 24 mM of proline. On the other hand, the lowest value (108.21) of F_0_ occurred in plants subjected to water salinity of 0.8 dS m^−1^ and a proline concentration of 1.5 mM. When comparing the maximum and minimum values obtained, an increase of 37.3% in the initial fluorescence was observed when plants were subjected to ECw of 3.6 dS m^−1^ and a proline concentration of 24 mM. 

**Figure 6:**
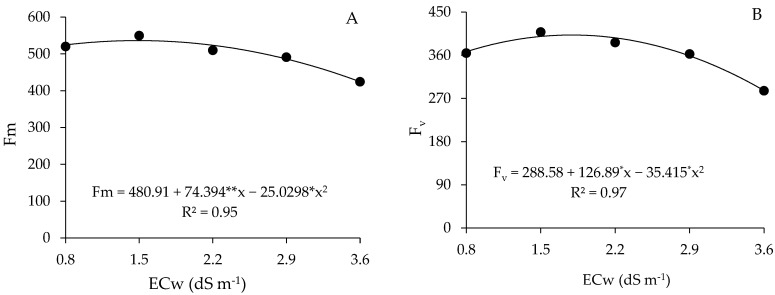
Maximum fluorescence (F_m_) (**A**) and variable (F_v_) (**B**) of guava plants cv. Paluma as a function of the levels of electrical conductivity of water (ECw) at 190 days after transplanting. ** and * significant at *p* ≤ 0.01 and *p* ≤ 0.05 according to the F test, respectively.

Maximum fluorescence (F_m_) showed a quadratic behavior, and the maximum estimated value (536.18) occurred in plants irrigated with 1.5 dS m^−1^ water, decreasing from this salinity level and reaching the lowest value (424.34) under ECw of 3.6 dS m^−1^ (Figure 6A). When comparing the F_m_ of plants subjected to the highest salinity (3.6 dS m^−1^) to the value of those cultivated under ECw of 0.8 dS m^−1^, a reduction of 20.85% was observed.

As observed for F_m_ (Figure 6A), variable fluorescence (F_v_) (Figure 6B) reached the maximum estimated value (402.23) in plants irrigated under water salinity of 1.8 dS m^−1^. When irrigated with water of 3.6 dS m^−1^, it resulted in the lowest Fv value (286.40), leading to a decrease of 28.8% when compared to the maximum values obtained in ECw of 1.8 dS m^−1^.

### 2.5. Growth Parameters

There was a significant effect between the factors (ECw × PROL) on crown diameter (D_crown_) and the absolute growth rate in stem diameter of guava plants (AGR_SD_) (Table 5). The water salinity levels significantly affected the stem diameter (SD), crown volume (V_crown_), and vegetative vigor index (VVI) at 360 DAT. The proline concentration had a significant effect on the relative growth rate in stem diameter (RGR_SD_) during the period from 190 to 360 days after transplanting.

**Figure 7:**
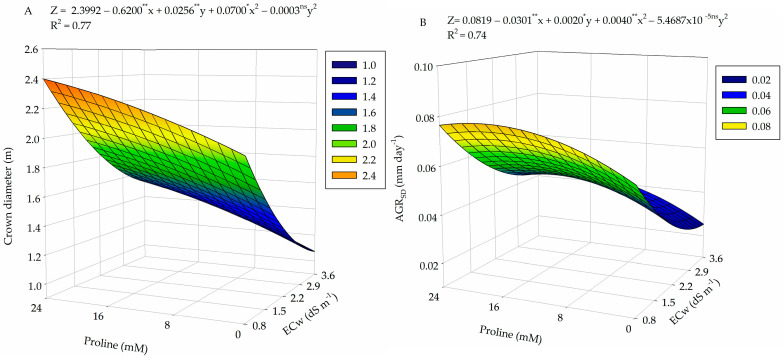
Crown diameter (**A**) and absolute growth rate in stem diameter (AGR_SD_) (**B**) of guava cv. Paluma as a function of the interaction between levels of electrical conductivity of water (ECw) and proline concentrations at 190 days after transplanting. X and Y: levels of electrical conductivity of water (ECw) and proline concentrations, respectively; ** and * significant at *p* ≤ 0.01 and *p* ≤ 0.05 and ^ns^ not significant (*p* > 0.05) according to the F test, respectively.

For crown diameter (D_crown_), the highest value (2.39 m) was observed in plants subjected to irrigation with ECw of 0.8 dS m^−1^ and foliar application of proline at a concentration of 24 mM, promoting an increase of 1.18 m (15.84%) when compared to plants under the control treatment (0.0 mM) and irrigated with the water salinity level of 3.6 dS m^−1^ (Figure 7A). Meanwhile, irrigation with 3.6 dS m^−1^ water and application of proline at a concentration of 0.0 mM resulted in the lowest D_crown_ (1.07 m). 

For the absolute growth rate in stem diameter (AGR_SD_) (Figure 7B), it was observed that irrigation with ECw of 0.8 dS m^−1^ associated with foliar application of proline at the estimated concentration of 18 mM resulted in the highest values (0.0784 mm day^−1^). When comparing the AGR_SD_ of plants that did not receive foliar application of proline and were irrigated with water of 0.8 dS m^−1^, whose estimated value was 0.0603, an increase of 23.08% was observed. Meanwhile, plants that did not receive foliar application of proline and were irrigated with ECw of 3.6 dS m^−1^ obtained the lowest value found (0.0253 mm day^−1^), which indicates a gain of 67.7% when compared with the maximum values obtained. 

**Figure 8:**
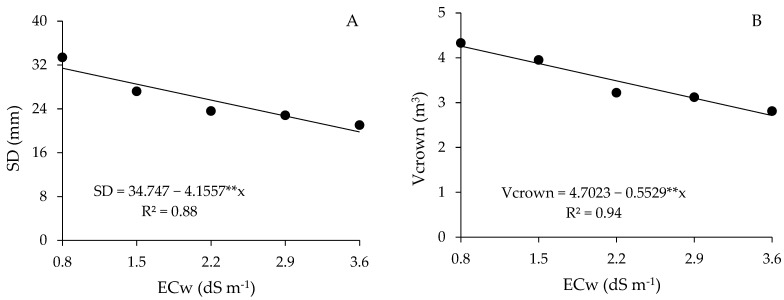

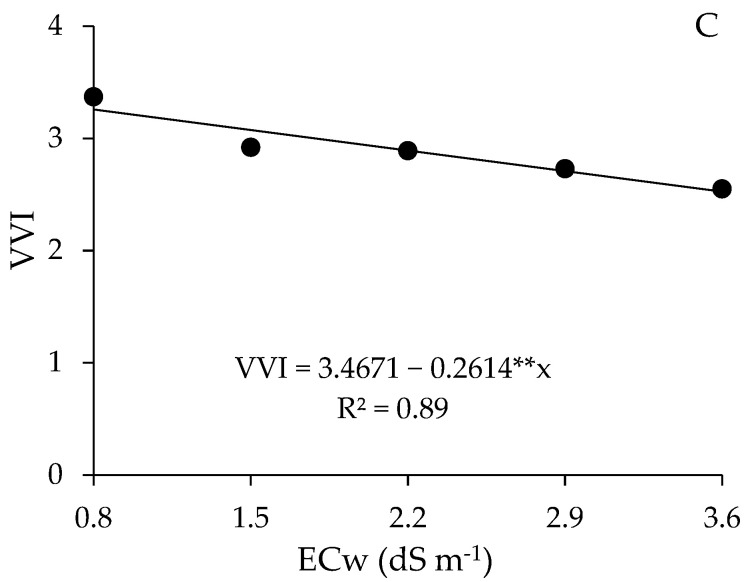
Stem diameter (SD) (**A**), crown volume (V_crown_) (**B**), and vegetative vigor index (VVI) (**C**) of guava plants cv. Paluma as a function of the levels of electrical conductivity of the water (ECw) at 190 days after transplanting. ** significant at *p* ≤ 0.01 according to the F test.

The increase in irrigation water salinity reduced the growth in stem diameter of guava plants (Figure 8A) by 11.96% per unit increment of ECw. When comparing the SD of plants cultivated with ECw 3.6 dS m^−1^ with that of plants subjected to water salinity of 0.8 dS m^−1^, a reduction of 37.03% (11.64 mm) was observed. 

Crown volume (V_crown_) (Figure 8B) and vegetative vigor index (VVI) (Figure 8C) also decreased linearly with the increase in the salinity levels of irrigation water by 11.76 and 7.54% per unit increase in ECw, respectively. When comparing the V_crown_ and VVI of plants irrigated with the highest salinity (3.6 dS m^−1^) with those obtained under the lowest ECw (0.8 dS m^−1^), reductions of 36.3% (1.55 m^3^) and 22.6% (0.73) were observed, respectively. 

**Figure 9:**
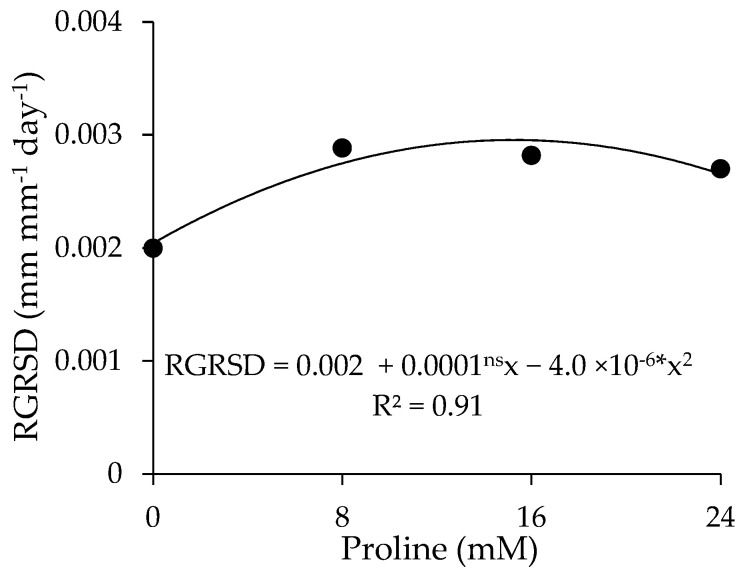
Relative growth rate in stem diameter (RGR_SD_) of guava cv. Paluma as a function of proline concentrations during the period from 190 to 360 days after transplanting. * significant at *p* ≤ 0.05 and ^ns^ not significant (*p* > 0.05) according to the F test, respectively.

Foliar application of proline favored the relative growth rate in stem diameter (RGR_SD_) of guava plants (Figure 9), with a maximum estimated value of 0.002625 mm mm^−1^ day^−1^ obtained at a concentration of 12.6 mM, representing an increase of 31.25% compared to plants that did not receive proline (0.0 mM). 

### 2.6. Principal Component Analysis

**Figure 10:**
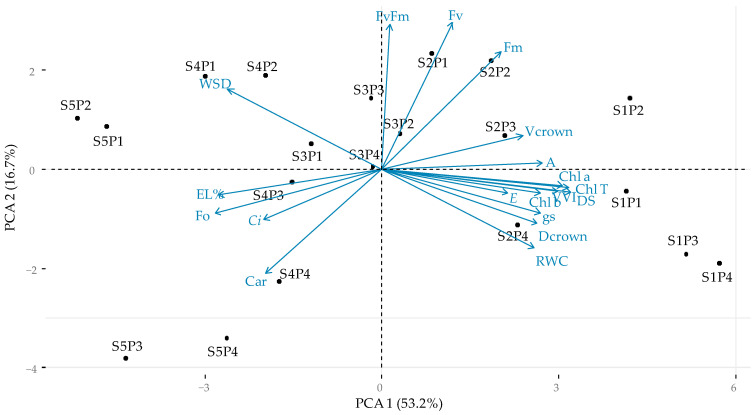
Principal component analysis (PCA) of correlation between physiological and growth variables of guava plants cv. ‘Paluma’ due to the interaction between the electrical conductivity levels of the irrigation water and proline concentrations at 390 days after transplanting. gs—stomatal conductance; E—transpiration; Ci—internal CO_2_ concentration; A—CO_2_ assimilation rate; RWC—relative water content; WSD—water saturation deficit; EL%—electrolyte leakage; Chl *a*—chlorophyll *a*; Chl *b*—chlorophyll *b*; Chl t—total chlorophyll; Car—carotenoids; F0—initial fluorescence; Fv—variable fluorescence; F_m_—maximum fluorescence; F_v_/F_m_—quantum efficiency of photosystem II; SD—stem diameter; V_crown_—crown volume; VVI—vegetative vigor index; D_crown—_crown diameter; absolute growth rate (AGR_SD_) and relative growth rate (RGR_SD_); S1P1—0.8 dS m^−1^ and 0 mM; S1P2—0.8 dS m^−1^ and 8 mM; S1P3—0.8 dS m^−1^ and 16 mM; S1P4—0.8 dS m^−1^ and 24 mM; S2P1—1.5 dS m^−1^ and 0 mM; S2P2—1.5 dS m^−1^ and 8 mM; S2P3—1.5 dS m^−1^ and 16 mM; S2P4—1.5 dS m^−1^ and 24 mM; S3P1—2.2 dS m^−1^ and 0 mM; S3P2—2.2 dS m^−1^ and 8 mM; S3P3—2.2 dS m^−1^ and 16 mM; S3P4—2.2 dS m^−1^ and 24 mM; S4P1—2.9 dS m^−1^ and 0 mM; S4P2—2.9 dS m^−1^ and 8 mM; S4P3—2.9 dS m^−1^ and 16 mM; S4P4—2.9 dS m^−1^ and 24 mM; S5P1—3.6 dS m^−1^ and 0 mM; S5P2—3.6 dS m^−1^ and 8 mM; S5P3—3.6 dS m^−1^ and 16 mM; S5P4—3.6 dS m^−1^ and 24 mM.

The correlation between physiological and growth variables with saline levels and proline concentrations presented by principal component analysis (PCA) indicates a representation of 69.9% in the first two components (Figure 10). It is possible to highlight that component 1 (PCA1) is responsible for 53.2%, being associated with treatments S1P1, S1P2, S1P3, and S1P4, which presented scores of −4.0418, −4.0919, −5.0365, and −5.5716, respectively. When associated with the variables evaluated, it is observed that these treatments showed high affinity with Chl a, Chl b, Chl t, and SD, which reached scores of −0.8903, −0.8629, −0.9190, and −0.9311, respectively. Also highlighted is a strong relationship between treatments and gas exchange *gs*, *E*, and A, which obtained scores of −0.7771, −0.6201, and −0.7886, respectively.

It was found that the second component (PCA 2) provided a contribution of 16.7%, with a strong association of Fv, Fm, and Fv/Fm (0.6242, 0.8539, and 0.8448, respectively) with treatments S2P2, S2P1, and S3P3, which have score values of 2.1306, 2.2656, and 1.3930, respectively. It is also worth mentioning the existence of a strong relationship between the S4P4 treatment (−2.2126) and the Car variable (−0.6101). Treatments S5P3 and S5P4 presented a good contribution to PC2, with scores of −3.7088 and −3.3274; however, they demonstrated low affinity with the variables analyzed (Figure 10).

## 3. Discussion

Foliar application of proline was beneficial in maintaining the water status of guava plants, causing an increase in the relative water content, which indicates that its use increases the plant’s tolerance to salt stress, thus favoring the absorption of water and nutrients and making the plant able to maintain the turgidity of its tissues. This is due to the fact that proline stimulates greater antioxidant activity, allowing the plant to adjust osmotically to stress conditions [21].

Water saturation deficit (WSD) was reduced by the foliar application of 24 mM of proline up to 1.5 dS m^−1^, which may be associated with an acclimatization mechanism through the accumulation of organic solutes, such as the accumulation of this amino acid in the vacuole of the plants, promoting osmotic adjustment, which led to a lower water deficit in the leaf blade [22].

It is possible to observe that the increase in salinity up to 3.6 dS m^−1^ compromised the maintenance of water status in the leaf blade of guava plants, with the highest WSD and EL% being observed under this condition. This behavior results from the deleterious effects caused by salt stress induced by the reduction of the plant’s ability to absorb water due to the decrease in the osmotic potential in the soil [12]. 

In the gas exchange, it was observed that the increase in water salinity above 0.8 dS m^−1^ led to partial closure of the stomata, thus reducing *gs* and transpiration and, consequently, the CO_2_ assimilation rate of guava plants. This limiting effect caused by salt stress occurs because plants partially close their stomata in order to avoid the loss of water to the atmosphere and reduce the absorption of toxic ions, such as Na^+^ and Cl^−^, thus avoiding the dehydration of the guard cells [9,23], a mechanism triggered to promote the maintenance of tissue turgidity [24].

Despite the stomatal limitation, there was an increase in the internal CO_2_ concentration in the substomatal chamber, highlighting non-stomatal factors, such as the limitation of the enzymatic activity of RuBisCO [25]. This fact did not directly affect the photosynthetic activity, as there was a reduction in CO_2_ assimilation with the increase in salinity above 0.8 dS m^−1^, which may have occurred due to the water deficit caused by the reduction in the osmotic potential of the soil, resulting in damage to PSII and restricting the capacity of CO_2_ assimilation [26], which explains why the increase in the internal CO_2_ concentration did not lead to an increase in the photosynthesis of guava plants.

This limitation caused by salt stress in gas exchange has also been observed by Nobre [27] in guava, Figueiredo [28] in pomegranate seedlings, Souza [29] in West Indian cherry plants, and Capitulino [30] in soursop.

Under low-salinity conditions (0.8 dS m^−1^), foliar application of proline improved gas exchange, as concentrations of 18 and 24 mM stimulated *gs*, transpiration, internal CO_2_ concentration, the CO_2_ assimilation rate, and the instantaneous carboxylation efficiency of guava plants, whereas WUEi was higher in plants under ECw of 0.98 dS m^−1^ and a proline concentration of 10.5 mM. When accumulated in cells, proline acts in plant acclimatization by protecting macromolecules against tissue damage and denaturation and increasing stress tolerance with osmotic adjustment and control of redox potential in energy production [31]. In addition, the application of proline may have a safeguarding effect against oxidative damage to cell organelles, especially leaf mesophylls, thus protecting them from the risks of cytotoxicity in leaf tissues [32] and ultimately mitigating the effects of salinity on guava gas exchange. 

The occurrence of this beneficial effect through the foliar application of proline in attenuating the effects of stress conditions was observed by Santos [16] in sour passion fruit (*Passiflora edulis* Sims.), for which the application of up to 6.5 mM of proline stimulated gas exchange in plants under salt stress. In sugar apple (*Annona squamosa* L.), Torres [33] reported that the application of 10 mM of proline promoted an increase in gas exchange in plants subjected to water deficit. Zahedi [34] observed that the combination of 100 mM of proline with graphene oxide stimulated the activity of antioxidant enzymes, thus reducing the effect of salt stress on grapevine plants (*Vitis vinifera* L.)

Salt stress compromised the synthesis of photosynthetic pigments in guava plants, possibly due to the excess of salts accumulated in the plant tissues, thus inducing the occurrence of damage to chloroplasts and resulting in protein denaturation and destabilization of membranes due to the excessive accumulation of toxic ions, causing inhibition in the synthesis of photosynthetic pigments [35,36]. In addition, salt stress also stimulates the activity of the enzyme chlorophyllase, which acts on the degradation of chlorophyll pigment molecules [37].

On the other hand, the application of proline was beneficial in the synthesis of chlorophyll, and increases in the contents of chlorophyll *a*, chlorophyll *b*, and total chlorophyll were observed with the increase in proline concentration. The increase in chlorophyll synthesis may be related to the fact that proline is an antioxidant that interacts with several enzymes, preserving and supporting the activity of proteins, as well as being involved in the regulation of genes that act in the biosynthesis of chlorophyll [38,39]. In addition, proline is closely linked to the increase in the synthesis of aminolevulinic acid, a precursor molecule of chlorophyll synthesis [40], and it participates as a constituent of several structuring proteins necessary for the synthesis and activation of chlorophyll [41], thus reducing the activity of the enzyme chlorophyllase, which acts on the degradation of photosynthetic pigments [42].

Similar results have been observed by Santos [16] in sour passion fruit irrigated with saline waters and subjected to proline concentrations, as foliar application of proline increased the synthesis of photosynthetic pigments, and by Zahedi [34] in grapevine plants, for which the combination of proline and graphene oxide promoted an increase in the synthesis of photosynthetic pigments.

In relation to the synthesis of carotenoids, the increase in water salinity and proline concentrations promoted an increase in their synthesis, which may be associated with the fact that carotenoids are responsible for the photoprotection of photosynthetic membranes, acting as an accessory pigment that helps in the dissipation of the excited state of chlorophyll and the neutralization of ROS [43]. Thus, foliar application of proline stimulates the activity of the antioxidant system against oxidative damage due to the ability to eliminate ROS from the cell [44], resulting in a higher carotenoid content in guava plants, even under conditions of salt stress.

The photochemical efficiency of guava plants was reduced by the increase in water salinity levels. However, an increase in F_0_ was observed as a function of water salinity, which is indicative of damage to the photosynthetic apparatus of the plants, as it is related to the loss of photochemical energy by chlorophylls in the antennae complexes of the photosystems [45]. In addition, F_v_ and F_m_ were reduced by salt stress, which implies the correct functioning of the reaction centers of PSII, which reduce its maximum capacity due to the reduction of quinone (QA) by the electrons transferred from P680, thus indicating a reduction in the activity of chlorophyll *a* [46]. 

The effect of reduction in gas exchange and photochemical efficiency was reflected in the inhibition of the growth of guava plants under salt stress. This is a consequence of the restriction in the absorption of water and nutrients caused by the decrease in the osmotic potential that induces partial closure of the stomata, leading to a reduction in the production of photoassimilates, and, combined with the accumulation of Na^+^ and Cl^−^, resulting in the decrease in pectin crosslinking, possibly produced by calcium deficiency. As a result, there is a loss of the turgor pressure of the cell in cell expansion and division, which directly interferes with plant growth [47,48]. In addition, salt stress affects the synthesis of structural constituents of the cell wall, such as suberin, lignin, and polysaccharides, which are related to plant growth [49].

It is worth pointing out that under conditions of low salinity (0.8 dS m^−1^), foliar application of proline at concentrations of 24 and 18 mM stimulated the growth in stem diameter and the absolute growth rate, which indicates the occurrence of a beneficial effect of proline on the growth of guava plants. The increase in growth may be related to the fundamental physiological and biochemical functions that proline exerts in plants, such as in the oxidative pathway of pentose phosphate, generating NADP^+^ in the cytosol, in addition to being a primary component of some important proteins in plant growth [21]. 

The principal component analysis shows that the foliar application of proline provided beneficial effects on the synthesis of photosynthetic pigments and gas exchange in guava, especially under low-salinity conditions (0.8 dS m^−1^). Still, according to the PCA, it is possible to highlight that the photochemical efficiency, represented by the variable and maximum fluorescence indices and the quantum yield of photosystem II, also benefited from the application of up to 16 mM of proline. It is worth noting that some authors classify guava as moderately tolerant to salinity [50], while others classify it as sensitive [12]. Thus, the foliar application of proline at the tested levels is an alternative option for the cultivation of guava cv. Paluma under conditions of moderate salinity, as our results demonstrated improvements in the physiological parameters evaluated.

## 4. Materials and Methods

### 4.1. Experimental Area Location and Characterization

**Figure 11:**
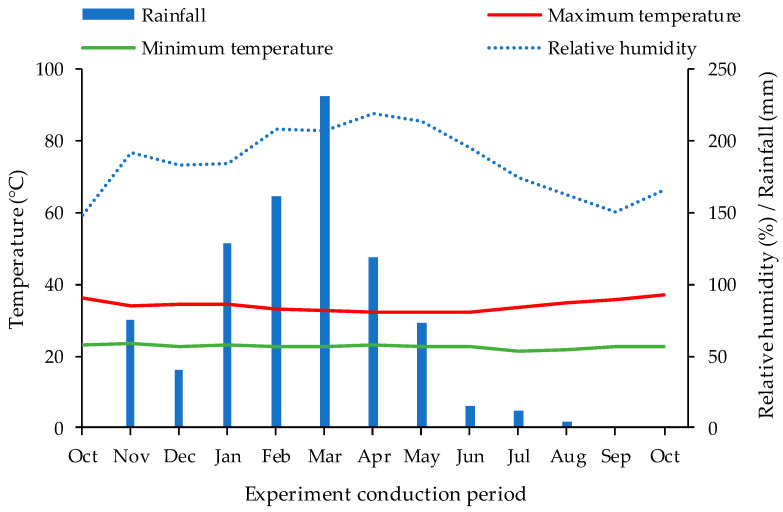
Maximum and minimum temperature and relative humidity data during the experimental period.

The experiment was conducted from October 2022 to October 2023 under field conditions at the ‘Rolando Enrique Rivas Castellón’ Experimental Farm, belonging to the Center for Science and Agrifood Technology (CCTA) of the Federal University of Campina Grande (UFCG), located in the municipality of São Domingos, Paraíba, Brazil, located at the coordinates 06°48′50″ latitude (S) and 37°56′31″ longitude (W), at an altitude of 190 m. During the experimental period, relative humidity and temperature (maximum and minimum) data were collected through the weather station of São Gonçalo, as presented in Figure 11.

### 4.2. Experimental Design and Treatments

The experiment was set up in a randomized block design, with the treatments arranged in a 5 × 4 factorial scheme, referring to five levels of electrical conductivity of water (ECw) (0.8, 1.5, 2.2, 2.9, and 3.6 dS m^−1^) and four concentrations of proline (0, 8, 16, and 24 mM) with three replicates, with the experimental plot consisting of one plant, totaling 60 experimental units. ECw levels were established based on a study conducted by Xavier [36] with guava cv. Paluma in the seedling formation stage. Proline concentrations were based on the results of the study conducted by Lima [51] with All Big bell pepper crop.

The guava cultivar Paluma was used in the present study, which stands out for its vigor and earliness, and whose propagation by cuttings results in flowering at 6 or 7 months of age after planting in the definitive location. The fruits are pyriform with smooth skin, and, when thinned, they can reach more than 500 g, especially in the first production cycles [52].

The seedlings were obtained from a commercial nursery accredited by the National Registry of Seeds and Seedlings located in the District of São Gonçalo, Sousa, PB, cultivated in black polyethylene bags with dimensions of 10 × 20 cm and a volumetric capacity of 0.5 L, and produced using the cutting technique.

### 4.3. Experiment Setup and Conduct

Guava plants were grown in plastic pots adapted as drainage lysimeters with 100 L capacity. Two holes were equidistantly made at the bottom of the lysimeters and connected to two 4 mm diameter plastic hoses, which were used for drainage and leaching of salts, and these drains were connected to 2 L plastic bottles. Inside of the lysimeters, a geotextile and a 0.5 kg layer of crushed stone were placed first, and then the lysimeters were filled with 110 kg of soil.

The soil used in the experiment was a *Neossolo Flúvico Ta Eutrófico típico* (Ultisol), with a loam texture, collected at a 0–30 cm depth from the experimental farm, belonging to the Center for Science and Agrifood Technology (CCTA) in São Domingo, PB. Chemical and physical attributes of the soil (Table 6) were determined according to the methodology of Teixeira [53].

The seedlings were transplanted to the lysimeters when the plants reached 150 days after propagation, a height of 32 cm, and a stem diameter of 7 mm. Soil moisture was raised to the level corresponding to field capacity using water with ECw of 0.8 dS m^−1^ up to 40 days after transplanting (DAT) to promote acclimation to the field conditions.

### 4.4. Preparation of Waters and Irrigation Management

The different levels of electrical conductivity of the irrigation water were prepared by diluting NaCl in water from the treatment of lowest salinity (0.8 dS m^−1^), from the supply system of the experimental farm, from an artesian well, following the relationship between ECw and the concentration of salts [54] according to Equation (1).

(1) Q≅10×ECw

where

*Q* = sum of cations (mmol_c_ L^−1^);

ECw = electrical conductivity after deducting ECw of water from the supply system (dS m^−1^).

After the acclimatization period of the plants in the lysimeters at 40 days after transplanting (DAT), irrigation began to be performed with the waters of the different salinity levels, and the volume of water was applied according to the water needs of the plants, determined by the water balance, according to Equation (2).

(2) $$VI=\frac{\left(Va-Vd\right)}{\left(1-LF\right)}$$

where

*VI* = volume of water to be used in the irrigation event (mL);

*Va* = volume of water applied in the previous irrigation event (mL);

*Vd* = volume of water drained (mL);

*LF* = leaching fraction (0.10) applied at 15-day intervals.

### 4.5. Preparation and Application of Proline Concentrations

Proline concentrations were prepared in each application event through dilution in distilled water, and 2 mL of Tween 80^®^ adjuvant solution was added to facilitate the fixation of the solution on the leaves. Applications were carried out monthly using a manual sprayer (Lynus PL-2A) with an adjustable 1 cm conical metal nozzle at an operating pressure of 64 Psi from 5 p.m. due to the lower temperature. A physical structure of polypropylene plastic tarpaulin was used to prevent the drift from affecting plants of another treatment. In total, an average volume of 331.73 mL per plant of the solutions with the different concentrations of proline was applied.

### 4.6. Crop Management

Fertilization with nitrogen (N), phosphorus (P), and potassium (K) was carried out according to the recommendation of Cavalcanti [55], considering the nutritional requirements of the crop and the contents of the elements in the soil. The sources used were urea (45% N), potassium sulfate (50% K_2_O), and monoammonium phosphate (50% P_2_O_5_ and 11% N). Fertilization started at 15 DAT and was split and applied via fertigation at 10-day intervals. Micronutrient fertilization was carried out weekly through the leaves, starting at 20 DAT, on the adaxial and abaxial surfaces, considering the nutritional requirements of the crop at the concentration of 1 g L^−1^ of Dripsol Micro^®^ (1.2% magnesium, 0.85% boron, 3.4% iron, 4.2% zinc, 3.2% manganese, 0.5% copper, and 0.06% molybdenum).

Guava plants were grown with a single stem until they reached 50 cm in height, and the apical bud was eliminated to stimulate the emergence of lateral bud shoots. From the appearance of the lateral branches, three to four well-located branches with 30 cm lengths, symmetrically and spirally distributed, were left. Subsequently, these branches were pruned when they reached 60 cm in length in order to stimulate the sprouting of secondary branches and to control lateral growth to form the basic structure of the crown and promote adequacy of the plants to the orchard spacing. During pruning, unwanted and poorly located branches were removed, especially those that were directed to the soil and to the interior of the crown.

The plants were trained by staking with a vertical trellis system by directing the branches toward the rows and the interrows. Phytosanitary control was carried out by manually eliminating weeds that appeared on the soil of the lysimeters and by using a hoe in the rows and interrows.

Pest control was carried out preventively upon the appearance of green aphid (*Myzus persicae*) and yellow beetle (*Costalimaita ferruginea vulgata)* through chemical intervention using Actara^®^ 250 WG (neonicotinoid–thiamethoxam) at a dose of 1 g for 10 L of water as recommended according to the package and Provado^®^ 200 SC (neonicotinoid–imidacloprid) at a dose of 2.5 mL for 10 L of water as recommended according to the package, respectively.

### 4.7. Variables Analyzed

#### 4.7.1. Relative Water Content, Electrolyte Leakage in the Leaf Blade, and Water Saturation Deficit

Analyses of relative water content, electrolyte leakage in the leaf blade, and water saturation deficit were performed at 360 days after transplanting. For the relative water content (RWC), fully expanded leaves were collected from the intermediate third of the branches, located in their terminal portion, and 8 leaf discs with 12 mm diameters were collected and their fresh mass (FM) was determined. Then, the samples were placed in plastic bags, immersed in distilled water, and stored for 24 h; after this period, excess water was removed with paper towels, and the turgid mass (TM) was obtained. Subsequently, the samples were dried in a forced-air circulation oven adjusted to a temperature of 65 °C until reaching constant mass to obtain the dry mass (DM). The samples were weighed on a 0.001 g precision semi-analytical scale.

RWC in the leaf blade was calculated according to Weatherley [56].

Electrolyte leakage in the leaf blade was determined by collecting eight leaf discs located in the middle third of the branch, in the terminal portion of the branch, and in the median region of the crown. After collection, the discs were immediately washed with distilled water in order to remove the contents of cells ruptured during collection and other electrolytes adhered to the leaves. After washing, the discs were dried on absorbent paper and placed in beakers containing 25 mL of distilled water at 25 °C for 24 h, where the initial electrical conductivity (Ci) was measured using an mCA 150 benchtop conductivity meter. Soon after, the beakers with the discs were placed in an incubator at 90 °C for 2 h, and then, with the temperature equilibrium, the final electrical conductivity (Cf) was measured. Electrolyte leakage (EL%) in the leaf blade was measured according to the methodology of Scotti-Campos [57].

Water saturation deficit is an indicator of the plant’s water balance, as it represents the amount of water it needs to reach saturation. In this context, WSD was determined according to the methodology described by Taiz [58].

#### 4.7.2. Gas Exchange

At 360 DAT, gas exchange was evaluated in the plants between 6:00 a.m. and 9:00 a.m. on the fourth fully expanded leaf from the apex to the base of the branch through determination of transpiration (E—mmol H_2_O m^−2^ s^−1^), stomatal conductance (gs—mol H_2_O m^−2^ s^−1^), CO_2_ assimilation rate (A—μmol CO_2_ m^−2^ s^−1^), intracellular CO_2_ concentration (Ci—μmol CO_2_ m^−2^ s^−1^), instantaneous carboxylation efficiency (CEi) (A/Ci) [(μmol CO_2_ m^−2^ s^−1^) (μmol CO_2_ mol^−1^]^−1^, and instantaneous water use efficiency (WUEi) [(μmol CO_2_ m^−2^ s^−1^) (mol H_2_O m^−2^ s^−1^)^−1^].

Readings were performed using a portable infrared carbon dioxide analyzer (IRGA) and a model LCPro+ Portable Photosynthesis System^®^ (ADC BioScientific Limited, United Kingdom) with irradiation of 1200 μmol photons m^−2^ s^−1^, air flow of 200 mL min^−1^, and atmospheric CO_2_ concentration.

#### 4.7.3. Photosynthetic Pigments

Contents of photosynthetic pigments were quantified at 306 DAT when leaf discs with an area of 3.14 cm^2^ were collected, placed in test vials, and wrapped in aluminum foil for protection against light. Then, 5 mL of dimethyl sulfoxide was added per sample. The protocol used for extraction and quantification was that described by Cruz [59]. In the laboratory, the vials were kept in the dark until completing 48 h from the moment of collection, after which the extracts were passed to the quartz cuvette and thus analyzed using the spectrophotometer at absorbances of 665, 649, and 480 nm. Contents of chlorophyll a (Chl a), chlorophyll b (Chl b), total chlorophyll (Chl total), and carotenoids (CAR) were determined by following the methodology of Wellburn [60].

#### 4.7.4. Photochemical Efficiency

Chlorophyll *a* fluorescence was measured through initial fluorescence (F_0_), maximum fluorescence (F_m_), variable fluorescence (F_v_), and quantum yield of PSII (F_v_/F_m_) in leaves pre-adapted to the dark using leaf clips for 30 min in the median leaf from the intermediate productive branch of the plant so as to ensure that all primary acceptors were oxidized, i.e., the reaction centers were open, using an OS5p pulse-modulated fluorometer from Opti Science.

#### 4.7.5. Growth

Growth was evaluated at 190 and 360 DAT based on stem diameter (SD), measured with a digital caliper at 3 cm height from the plant collar, crown diameter (D_crown_), obtained through the average of crown diameter observed in the row direction (RD) and interrow direction (IRD), crown volume (V_crown_), defined according to plant height (H), RD, and IRD, and the vegetative vigor index (VVI), obtained according to Portella [61].

The absolute (AGR_SD_) and relative (RGR_SD_) growth rates in stem diameter were determined according to the methodology of Benincasa [62].

### 4.8. Statistical Analysis

The data were analyzed for normality (Shapiro–Wilk test) and then subjected to analysis of variance (F test) at 0.05 and 0.01 probability levels; in cases of significance, linear and quadratic regression analysis was performed for the levels of electrical conductivity of irrigation water and proline concentrations using the statistical software SISVAR—ESAL version 5.7 [63]. To evaluate the correlations between physiological and growth parameters and the salinity levels of the irrigation water and proline concentrations, a principal component analysis (PCA) was performed.

## 5. Conclusions

Irrigation water salinity compromises the growth and development of guava plants, being one of the main problems faced by producers in the semi-arid region of northeast Brazil. In this study, exogenous application of proline proved to be a viable and low-cost alternative to reducing the deleterious effects of salt stress, thus promoting improvements in gas exchange, pigment synthesis, and growth of guava plants at 360 days after transplanting. However, further research is still needed in order to elucidate the biochemical effects that proline provides as an attenuator of the harmful effects of salt stress. This demonstrates that its use can become an important tool for producers in semi-arid regions, such as the Brazilian northeast.

## Figures and Tables

**Table 1:** Summary of the analysis of variance for the relative water content (RWC), water saturation deficit (WSD), and electrolyte leakage (EL%) in the leaf blade of guava plants cv. Paluma cultivated under different electrical conductivities of irrigation water (ECw) and proline concentrations (PROL) at 360 days after transplanting.

Source of Variation	DF	Mean Squares		
		RWC	WSD	EL%
Salinity levels (ECw)	4	1022 **	1033 **	746 **
Linear regression	1	3899 **	3934 **	2886 **
Quadratic regression	1	138 ^ns^	144 ^ns^	73 *
Proline (PROL)	3	998 **	992 **	112 **
Linear regression	1	2500 **	2473 **	16 ^ns^
Quadratic regression	1	491 **	500 **	164 **
Interaction (ECw × PROL)	12	126 **	127 **	63 **
Blocks	2	90 ^ns^	89 ^ns^	14 ^ns^
Residual	38	45	45	17
CV (%)		8.51	31.80	14.58

DF—Degrees of freedom; CV (%)—coefficient of variation; ** significant at 0.01 probability level; * significant at 0.05% probability level; ^ns^ not significant.

**Table 2:** Summary of the analysis of variance for stomatal conductance (gs), transpiration (E), internal CO_2_ concentration (Ci), CO_2_ assimilation rate (A), instantaneous water use efficiency (WUEi), and instantaneous carboxylation efficiency (CEi) of guava plants cv. Paluma cultivated under different electrical conductivities of irrigation water (ECw) and proline concentrations (PROL) at 360 days after transplanting.

Source of Variation	DF	Mean Squares					
		*gs*	*E*	*Ci*	*A*	*WUEi*	*CEi*
Salinity levels (ECw)	4	0.044 **	4.87 **	12106 **	196.55 **	4.67 ^ns^	0.0141 **
Linear regression	1	0.124 **	17.34 **	46256 **	775.71 **	9.22 ^ns^	0.0550 **
Quadratic regression	1	0.039 **	1.64 ^ns^	125 ^ns^	0.43 ^ns^	6.94	0.0010 ^ns^
Proline (PROL)	3	0.005 **	0.42 ^ns^	6356 **	45.22 *	0.44 ^ns^	0.0018 ^ns^
Linear regression	1	0.015 **	1.09 ^ns^	5534 **	85.86 **	0.30 ^ns^	0.0008 ^ns^
Quadratic regression	1	0.0004 ^ns^	0.17 ^ns^	9238 **	26.00 ^ns^	0.61 ^ns^	0.0045 **
Interaction (ECw × PROL)	12	0.005 **	2.29 *	3295 **	33.62 **	8.38 **	0.0020 *
Blocks	2	0.004 *	1.39 ^ns^	229 ^ns^	7.11 ^ns^	1.32 ^ns^	0.0006 ^ns^
Residual	38	0.001	0.87	769	12.09	2.50	0.0008
CV (%)		26.17	27.42	26.17	25.76	14.64	36.12

DF—Degrees of freedom; CV (%)—coefficient of variation; ** significant at 0.01 probability level; * significant at 0.05% probability level; ^ns^ not significant.

**Table 3:** Summary of the analysis of variance for chlorophyll *a* (Chl *a*), chlorophyll *b* (Chl *b*), Chl *a*/Chl *b* ratio (Chl *a*/Chl *b*), total chlorophyll (Chl *T*), and carotenoids (Car) of guava plants cv. Paluma cultivated under different electrical conductivities of irrigation water (ECw) and proline concentrations (PROL) at 360 days after transplanting.

Source of Variation	DF	Mean Squares				
		Chl *a*	Chl *b*	Chl *a*/Chl *b*	Chl *T*	Car
Salinity levels (ECw)	4	74.34 **	8.76 **	0.45 ^ns^	132.43 **	6.00 **
Linear regression	1	277.3 **	33.88 **	0.04 ^ns^	504.99 **	23.46 **
Quadratic regression	1	15.16 **	0.02 ^ns^	1.46 *	14.03 *	0.10 ^ns^
Proline (PROL)	3	17.09 **	2.36 **	1.73 **	26.46 **	4.70 **
Linear regression	1	41.21 **	4.60 **	0.03 ^ns^	73.22 **	13.79 **
Quadratic regression	1	5.11 ^ns^	2.47 *	4.48 **	0.47 ^ns^	0.00008 ^ns^
Interaction (ECw × PROL)	12	2.97 ^ns^	0.60 ^ns^	0.89 ^ns^	3.84 ^ns^	1.15 **
Blocks	2	0.21 ^ns^	0.26 ^ns^	0.11 ^ns^	0.02 ^ns^	0.53
Residual	38	2.12	0.46	0.29	3.03	0.40
CV (%)		13.13	15.92	20.50	11.32	13.15

DF—Degrees of freedom; CV (%)—coefficient of variation; ** significant at 0.01 probability level; * significant at 0.05% probability level; ^ns^ not significant.

**Table 4:** Summary of the analysis of variance for initial (F_0_), maximum (F_m_), variable (F_v_) fluorescence, and quantum efficiency of photosystem II (F_v_/F_m_) of guava plants cv. Paluma cultivated under different electrical conductivities of irrigation water (ECw) and proline concentrations (PROL) at 360 days after transplanting.

Source of Variation	DF	Mean Squares			
		F_0_	F_m_	F_v_	F_v_/F_m_
Salinity levels (ECw)	4	5073 **	26,326 **	25,561 *	0.014 ^ns^
Linear regression	1	20,254 **	75,075 **	49,227 *	0.006 ^ns^
Quadratic regression	1	3.72 ^ns^	25,271 *	50,596 *	0.051 *
Proline (PROL)	3	502 ^ns^	4951 ^ns^	16,240 ^ns^	0.023 ^ns^
Linear regression	1	1310 *	8883 ^ns^	26,498 ^ns^	0.030 ^ns^
Quadratic regression	1	176 ^ns^	2554 ^ns^	5890 ^ns^	0.005 ^ns^
Interaction (ECw × PROL)	12	581 *	3874 ^ns^	6013 ^ns^	0.009 ^ns^
Blocks	2	383 ^ns^	1649 ^ns^	3257 ^ns^	0.003 ^ns^
Residual	38	256	6021	7314	0.009
CV (%)		11.54	15.55	23.65	13.78

DF—Degrees of freedom; CV (%)—coefficient of variation; ** significant at 0.01 probability level; * significant at 0.05% probability level; ^ns^ not significant.

**Table 5:** Summary of the analysis of variance for stem diameter (SD), crown volume (V_crown_), vegetative vigor index (VVI), and crown diameter (D_crown_) at 360 days after transplanting (DAT) and absolute growth rate (AGR_SD_) and relative growth rate (RGR_SD_) in stem diameter of guava cv. Paluma cultivated under different electrical conductivities of irrigation water (ECw) and proline concentrations (PROL) during the period from 190 to 360 days after transplanting (DAT).

Source of Variation	DF	Mean Squares					
		SD	V_crown_	VVI	D_crown_	AGR_SD_	RGR_SD_
Salinity levels (ECw)	4	287.47 **	4.75 **	1.11 **	1.47 **	0.0021 **	0.000007 ^ns^
Linear regression	1	1016.40 **	17.91 **	4.00 **	5.69 **	0.0078 **	0.000003 ^ns^
Quadratic regression	1	115.46 **	0.48 ^ns^	0.14 ^ns^	0.19 ^ns^	0.0006 ^ns^	0.000000 ^ns^
Proline (PROL)	3	8.60 ^ns^	0.61 ^ns^	0.07 ^ns^	0.58 **	0.0011 *	0.000003 *
Linear regression	1	25.28 ^ns^	1.42 ^ns^	0.14 ^ns^	1.69 **	0.0021 *	0.000003 ^ns^
Quadratic regression	1	0.03 ^ns^	0.35 ^ns^	0.0001 ^ns^	0.01 ^ns^	0.0012 ^ns^	0.000004 *
Interaction (ECw × PROL)	12	14.93 ^ns^	1.40 ^ns^	0.30 ^ns^	0.44 **	0.0007 *	0.000002 ^ns^
Blocks	2	12.85 ^ns^	0.09 ^ns^	0.07 ^ns^	0.05 ^ns^	0.0002 ^ns^	0.000002 ^ns^
Residual	38	8.57	0.83	0.16	0.06 ^ns^	0.0003	0.000008
CV (%)		11.44	26.14	14.20	15.22	35.58	34.50

DF—Degrees of freedom; CV (%)—coefficient of variation; ** significant at 0.01 probability level; * significant at 0.05% probability level; ^ns^ not significant.

**Table 6:** Chemical and physical attributes of the soil used during the experiment before the application of the treatments.

**Chemical characteristics**									
pH (H_2_O) 1:2.5	OM dag kg^−1^	P (mg kg^−1^)	K^+^	Na^+^	Ca^2+^	Mg^2+^	Al^3+^ +H^+^	ESP (%)	ECse (dS m^−1^)
			(cmol_c_ kg^−1^)						
7.19	1.40	59.5	0.49	0.07	4.70	3.63	0	0.79	0.58
**Physical characteristics**									
Particle-size fraction (g kg^−1^)			Textural class	Moisture (kPa)		AW	Total porosity	BD	PD
Sand	Silt	Clay		33.43 *	1519.8 **			(kg dm^3^)	
	
				...........	dag kg^−1^	..........	%		
735.1	201.4	60.30	SL	15.78	6.41	9.37	55.05	1.20	2.67

OM—Organic Matter: Walkley–Black Wet Digestion; Ca^2+^ and Mg^2+^ extracted with 1 M KCl at pH 7.0; Na^+^ and K^+^ extracted with 1 M NH_4_Oac at pH 7.0; Al^3+^ and H^+^ extracted with 0.5 M CaOAc at pH 7.0; ESP—exchangeable sodium percentage; ECse—electrical conductivity of saturation extract; SL—sandy loam; AW—available water; BD—bulk density; PD—particle density; *—field capacity; **—wilting point.

## Data Availability

Data are contained within the article.
